# Kaposi Sarcoma among HIV Infected Patients in Lagos University Teaching Hospital, Nigeria: A 14-Year Retrospective Clinicopathological Study

**DOI:** 10.1155/2016/9368023

**Published:** 2016-03-13

**Authors:** Olakanmi Akinde, Omobolade Obadofin, Titilope Adeyemo, Oladipo Omoseebi, Nzechukwu Ikeri, Ikechukwu Okonkwo, Olatunji Afolayan

**Affiliations:** ^1^Department of Anatomic and Molecular Pathology, Lagos University Teaching Hospital, PMB 12003, Idi-Araba, Lagos, Nigeria; ^2^Department of Hematology, Lagos University Teaching Hospital, PMB 12003, Idi-Araba, Lagos, Nigeria; ^3^Department of Surgery, Lagos University Teaching Hospital, PMB 12003, Idi-Araba, Lagos, Nigeria

## Abstract

*Background.* Despite the increased incidence of Kaposi sarcoma (KS) resulting from the Human Immunodeficiency Virus/Acquired Immunodeficiency Syndrome (HIV/AIDS) pandemic, there is still significant underreporting of KS in this environment.* Objectives.* This study was aimed at determining the incidence and clinicopathologic patterns of KS among HIV infected patients in Lagos University Teaching Hospital (LUTH), Nigeria, over a 14-year period: January 2000 to December 2013.* Methodology.* The materials for this study included patients' hospital clinical files, duplicate copies of histopathologic reports, and tissue blocks and corresponding archival slides in the Anatomic and Molecular Pathology Department and the HIV/AIDS unit of the Department of Haematology.* Results.* Within the study period, 182 cases of KS were diagnosed, accounting for 1.2% of all patients managed for HIV/AIDS and 2.99% of solid malignant tumours. The male-to-female ratio and modal age group were 1 : 1.3 and 5th decade, respectively. Most cases (90%) had purely mucocutaneous involvement with the lower limb being the commonest site (65.8%). The majority of lesions were plaques (65.8%). Vascular formation was the predominant histologic type seen (43.5%).* Conclusion.* KS in Lagos followed the same epidemiologic trend as other centers in Nigeria, with an increasing incidence in this era of HIV/AIDS.

## 1. Introduction

Kaposi sarcoma (KS) is a vascular lesion of low-grade malignant potential that presents most frequently with skin lesions. It is the most common AIDS associated malignancy, and human herpes virus type 8 (HHV8) plays a significant role in its aetiopathogenesis [1, 2].

A number of studies have reported both a change in the pattern and a dramatic increase in the incidence of KS as a consequence of the increased incidence of AIDS [3, 4]. The pattern of the disease has shown variation with location and time [5, 6]. For example, the pattern of KS among the Caucasians differs significantly from that in tropical Africa in terms of the frequency, risk, sex and age distribution, and trend [7–15]. Even within the same country, the incidence of KS is not uniform. A previous study on malignant skin tumours carried out by Adeyi and Banjo in the center of this study reported only 8 cases of Kaposi sarcoma over 6 years (1990–1995) [16]. In Ibadan, southwest Nigeria, Gana and Ademola reported KS to constitute 8.3% (41 cases) of malignant skin tumours over 20 years (1981–2000) [17]. Oluwasanmi and Osunkoya recorded fewer cases at the same center in 1969 [18]. In a recent study of vasoformative neoplasms by Abudu et al. in Sagamu, no case of KS was seen over a 5-year period (2004–2008) [19]. In contrast to these findings in southwestern Nigeria, there is a relatively higher incidence of the tumour in the southeastern part of the country. Asuquo et al. reported KS to be the commonest skin cancer in Calabar where 11 cases were seen in two years (2005 and 2006). This was at variance with earlier findings reported in the same center where it was second to squamous cell carcinoma with 17 cases seen over 5 years (2000–2004) [20]. Also, in Benin, the same region of the country as Calabar, Onunu et al. reported 31 cases over 5 years (July 2000 to June 2005) [4].

Most reports from the northern part of Nigeria also indicate a change in the pattern of KS. In Jos, Mandong et al. reported 65 cases over 16 years (1987–2002) [21]. More recently, however, Mohammed et al. reported 32 cases from the same center over 5 years (2000–2004) [3]. Mbah et al. reported only 27 cases in 11 years (1994–2004) in Sokoto [22]. However, Kagu et al. reported 20 cases in 2 years in Maiduguri (September 2003 to August 2005) [23].

There is high prevalence of HIV/AIDS in Lagos as in other parts of Nigeria. While it might be assumed that the pattern of KS in this center conforms to the pattern seen in other centers and African countries, it is still necessary to get recent and accurate information about the disease in the country, if meaningful strategies for treatment and prevention are to be made.

There has been evidence of an association of KS with second primary malignancies. Most often, patients with classic KS are at special risk of developing a malignancy of the lymphoreticular system [11]. In a study done by Safai, 37% of KS patients had other primary malignancies such as chronic lymphocytic lymphoma.

With improving care of people with HIV/AIDS and better management of related infections, especially tuberculosis, the longer survival of affected people in Nigeria will likely increase the burden of cancer as a clinical problem. However, reduction in the HIB/AIDS related KS may be an indicator of control of HIV/AIDS. The population's geographic and social diversity makes Nigeria a unique country for research on HIV/AIDS associated cancers. This can be embedded in programs targeting HIV/AIDS and other public health priorities. This study therefore was aimed at determining the incidence of KS among HIV infected patients in Lagos University Teaching Hospital. We also looked into the different clinical types of KS, their various histologic variants, and the impact of HIV/AIDS on the prevalence of KS.

## 2. Materials and Methods

The materials for this study were derived from patients' hospital clinical files and request forms, duplicate copies of histopathological reports that were issued within the study period, and tissue blocks and corresponding archival slides in the Anatomic and Molecular Pathology Department of LUTH and the HIV/AIDS unit of the Department of Haematology, LUTH. All records of clinically diagnosed KS and those with histologically confirmed cases of KS from 2000 to 2013 were analyzed for age, sex, histologic variant, and the sites of distribution as well as their HIV/AIDS status. The frequency of the tumour was calculated relative to HIV/AIDS burden as well as all malignancies diagnosed in the same period. Histological classification was based on the morphological appearance of the tumour. Data were presented in figures, percentages, and simple frequency tables. The analysis of data from cases without histologic diagnoses served as a limitation of the study.

## 3. Results

**Figure 1:**
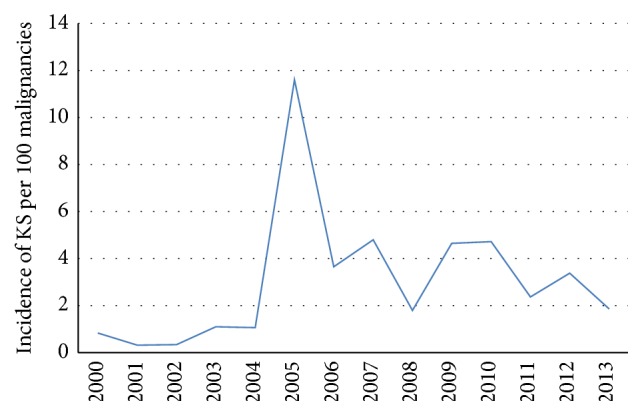
Trends in the incidence of KS from 2000 to 2013.

The relative frequency is shown in Table 1. One hundred and eighty-two cases of KS, composed of 115 and 67 clinically and histologically diagnosed cases, respectively, were seen over a period of 14 years. This constituted 1.2% of all the patients managed for HIV/AIDS (182 of 15,447 cases) and 2.99% of solid malignant tumours seen in the center during the period of the study. The proportion of KS among malignancies seen was low in the early years of the study but sharply increased in the latter period as shown in Figure 1.

**Figure 2:**
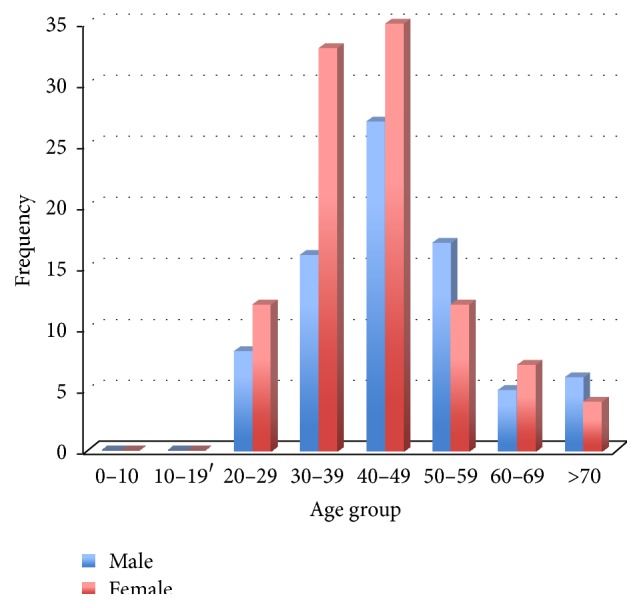
Age and sex distribution of KS.

Figure 2 shows the sex and age distribution of KS. Seventy-nine of the 182 cases (43.4%) were females, while 103 of the 182 (56.6%) were males. These represented 0.8% of females with HIV (79 of 10,227 cases) and 2.0% (103 of 5,220 cases) of HIV infected males. The male-to-female ratio was 1 : 1.3. There was a wide age range of presentation (1st to the 8th decades); however, the modal age group was the 5th decade (40–49 years), while the average age of incidence was about 45 years.

**Figure 3:**
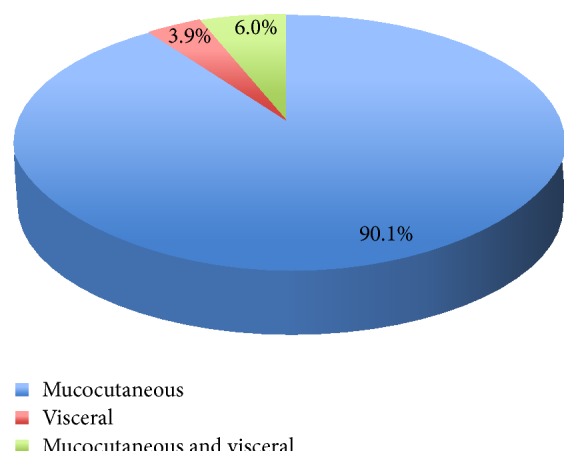
Anatomic distribution of KS lesions.

The distribution of KS by site is shown in Figure 3. One hundred and sixty-four cases (90.1%) had only mucocutaneous involvement (skin, conjunctiva, and oral mucosa) and 11 (6%) had mucocutaneous and visceral involvement, while 7 (3.9%) had purely visceral involvement (5 lungs, 2 GIT).

Table 2 shows the distribution of the mucocutaneous lesions. One hundred and eight (65.8%) of those with only cutaneous lesions had multiple sites involved, with the lower limbs being the most affected site. Thirty-six of the 56 (64.3%) patients with a single cutaneous site had lower limb involvement while 8 (14.3%), 7 (12.5%), and 5 (8.9%) cases had involvement of the head, trunk, and upper limbs, respectively.

The most common lesions on the skin were plaques. These were seen in 108 of the patients and, of these, 67 (62%) had clinically equivocal lesions but were confirmed by histology while the diagnosis of the remaining 41 (38%) cases was based on clinical assessments. 29 cases had multiple nodular lesions involving only the lower limbs while 27 patients had a mixture of lesions including plaques, nodules, and ulcers involving multiple sites, especially the lower limbs.

**Figure 4:**
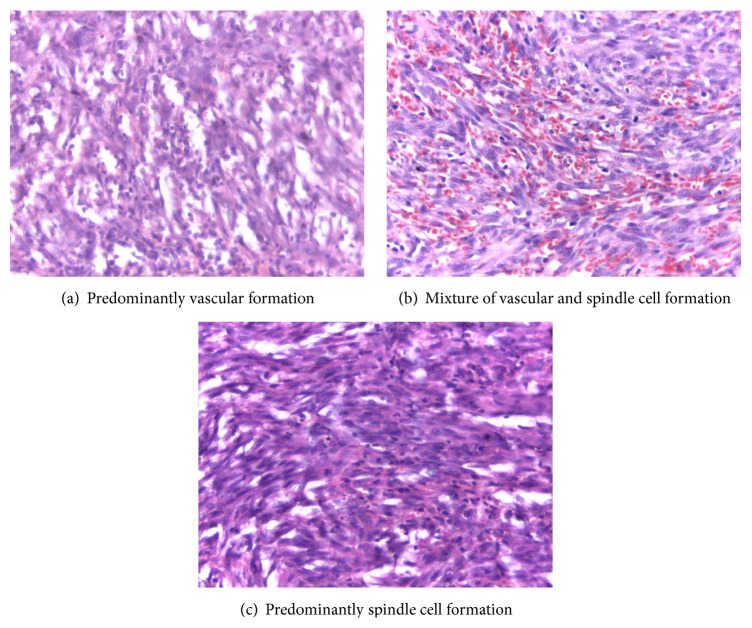
Histologic patterns of KS.

The histologic patterns are shown in Figures 4(a)–4(c). Of the 67 cases with histological diagnosis, two patterns were seen, predominantly spindle cell formations and vascular formations with frequencies of 17 (25.3%) and 29 (43.5%), respectively, while mixtures of the two formations were seen in 21 (31.2%) of the cases. Inflammatory response with granulation tissue formation was seen in 75% of the lesions irrespective of the histological type.

Three cases of childhood KS were seen among the histologically diagnosed cases: a 7-month-old boy with swellings on the head since birth, a 6-year-old girl with generalized nodular rashes, and a 10-year-old girl with generalized body rash.

## 4. Discussion

Only 182 cases of Kaposi sarcoma over a period of 14 years were recorded during the study period. This implies an average annual frequency of 13 cases. Lower incidence was earlier reported in the same center by Adeyi and Banjo who reported 8 cases of cutaneous KS over 6 years (1990 and 1995) [16]. This finding is consistent with data from different parts of the country with an upward trend in the incidence of KS. Some centers have reported KS as the most frequent cutaneous cancer [3, 24]. However, literature from other centers across Nigeria shows KS to be of low incidence relative to the burden of other cancers such as cancer of the breast, cervix, colon, prostate, and liver across the country [25, 26]. This is in contrast to the findings in other parts of Africa such as Uganda [6, 9], Zimbabwe [10, 14], Zambia [13], Rwanda [11], and South Africa [5], where KS has become the major cause of cancer morbidity and mortality.

Kaposi sarcoma was not featured as one of the common cancers in Nigeria before the HIV/AIDS pandemic. In fact, most records at different cancer registries did not show cases of KS in their locality, probably because of its low occurrence. There was no case of KS in the records of cancer registry, UCH, Ibadan, before 1980 but between 1981 and 2000, 30 cases of KS were recorded in the center [17, 26]. Although previous underreporting of cases of Kaposi sarcoma may be playing an important role in the changing pattern of the tumour in the country, it is obvious that HIV/AIDS has contributed to the changing pattern of the disease across the country. The majority of the cases in our study (161 cases, 88.5%) were associated with HIV infection. In the remaining 21 cases (11.5%), there was no information as to the HIV status of the patients as the tissues biopsies of these were referred for histopathologic diagnosis from a private hospital. With the availability of several alternative public and private health care facilities in Lagos and across the country, a large number of people with Kaposi sarcoma might seek medical attention at these facilities. It is our view that this study, which is hospital based, rather than a population based survey and hence not a true picture of Kaposi sarcoma in Lagos state, will nonetheless add to the information from these other centers. As in the center of this study, many cases of KS are diagnosed clinically. This is partly due to the reluctance of many clinicians to carry out biopsies in HIV/AIDS patients for fear of possible self-injury and contraction of HIV infection. This will be one of the many other factors of KS underreporting.

Although there is no previous similar study in the center of this study on the incidence of KS before the advent of AIDS pandemic, this study agreed with similar studies from other centers within the country and other African countries that, with the advent of AIDS pandemic, there has been an increase in the incidence of KS [3, 24]. Previous studies by Oluwasanmi and Osunkoya in Ibadan [18] and Otu in Calabar [27] indicated that KS in Nigeria was not associated with HIV infection and that Kaposi sarcoma associated herpes virus (KSHV) was not endemic in this region. More recent studies, however, have indicated increased incidence and changing pattern of KS with the advent of HIV/AIDS pandemic [3, 24]. Studies have also implicated KSHV in the aetiopathogenesis of KS as this virus is readily found in all forms of KS. The virus is sexually transmitted and can also be transmitted through organ donation. In Africa, studies have shown a high rate of KSHV infection with the resultant increase in incidence of KS, which has become the most common cancer in sub-Saharan Africa. KSHV infection is thought to be lifelong so that a person infected with KSHV may develop KS years later if they develop HIV/AIDS or other forms of immunosuppressive disorders [28].

Seropositivity for KSHV in the general populace and among people infected with HIV is not known in this environment. This is an area for further study as the development of the tumour relates closely to the plasma titer value of the virus as well as the degree of immunosuppression as determined by the value of CD4^+^ T lymphocyte count.

Literature review revealed that the pattern of occurrence and trend of the disease differ from one country to another. In developed countries, for instance, AIDS associated KS has been seen most commonly among homosexual men, the incidence of which is declining. It is rarely seen among patients who have acquired HIV infection through heterosexual contact, intravenous drug use, or vertical transmission [29]. In our setting, HIV infection seems to be contracted and spread through heterosexual rather than homosexual means and intravenous drug abuse. The route of contact might not be relevant to the occurrence of KS as this is a consequence of the level of immunosuppression.

Three cases of histologically diagnosed childhood KS with HIV infection were seen. This low incidence may be due to the possibility that childhood KS is not common in Nigeria or that a long latent time is required for the development of KS, which may be also influenced by medication. However, one has to be sure that cases of paediatric KS are not being missed or wrongly diagnosed as vascular malformations, for example, haemangioma, granuloma pyogenicum, granulation tissue, popular urticaria, and inflammatory dermatoses. In Uganda, the incidence of childhood Kaposi sarcoma has risen more than 40-fold in the era of HIV/AIDS [30]. A high index of suspicion, biopsy for histology, and special tests like immunohistochemistry/molecular techniques to detect presence of HHV8-LANA-1 might be of help.

KS is not a clinical diagnosis as various lesions such as melanoma, mycoses, and Madura foot, which are also common on the leg, could mimic KS. Since these entities have also been reported in patients with HIV, obtaining a histologic confirmation of KS is important. However, this poses a significant financial constraint to many patients undergoing treatment for HIV in our environment. This lack of histologic confirmation in a proportion of cases served as a limitation for our study.

Histopathologists are familiar with the histological features of usual (typical) KS as it progresses from patch to plaque and finally nodular stages [31–33]. This morphologic spectrum of “usual” KS is common to classic, African endemic, transplant-associated, and acquired immune deficiency syndrome (AIDS) associated KS. However, studies have shown that KS exists in a wider histologic spectrum. These include anaplastic, lymphedematous, lymphangioma-like, lymphangiectatic telangiectatic, and bullous-like Kaposi sarcoma, as well as verrucous, keloidal, ecchymotic, intravascular, and granulomatous variants [34, 35].

In this study, the histological patterns of KS observed, as shown in Figure 4, were lesions with predominant vascular formation, lesions with predominant spindle cell formations, and lesions in which both vascular and spindle cell formation are prominent. These were seen in varying degrees in each of the lesions. However, depending on the clinical stage of the disease, a particular histologic type could predominate.

Predominantly vascular formation shown in Figure 4(a) was seen in 43.5% of the cases and formed majority of the cases. This picture is a feature of early patch stage of Kaposi sarcoma. Dilated thin walled vascular channels with jagged outlines were seen. These vessels may not contain red blood cells resembling lymphatic structures. The endothelial lining is not atypical. This type of formation may be confused with lymphangioma or haemangioma and this variant of KS should be subjected to immunohistochemistry for a definitive diagnosis. The cases with vascular and spindle cell formations (31.2%) (Figure 4(b)) were most easily diagnosed. They were commonly seen in the plaque and nodular stages, the stage in which many of the patients in this study presented. Cases with predominant proliferation of spindle cells (Figure 4(c)) can be wrongly diagnosed as fibrosarcoma, as some of the cells show nuclear atypia and increased mitoses. The presence of narrow slits containing red blood cells, however, will distinguish KS from fibrosarcoma.

Inflammatory response is commonly associated with ulcerated skin lesions and this was seen in 75% of cases irrespective of the pattern. Inflammatory response giving the appearance of granulation tissue can lead to misdiagnosis of KS especially in early lesions. In cases of KS, the endothelial cell lining is large and protrudes into the lumen and the inflammatory cell infiltrates are of varying intensity and are perivascular or diffusely disposed. Also, the presence of extravasated red blood cells and hemosiderin always indicates the possibility of early KS.

Failure to identify a given lesion as KS could lead to wrong or delayed diagnosis and hence inappropriate management. Immunohistochemistry should be utilized in controversial cases.

Majority of the cases of Kaposi sarcoma in this study were mucocutaneous and many of the cases were multifocal involving multiple sites (upper limbs, lower limbs, head region, and scrotum). In most of these cases, the lower limbs were mostly involved. This observation is in agreement with other reports on KS. One would expect more cases of KS in this setting to involve the viscera because of late presentation and association with HIV. In resource poor centers where sophisticated diagnostic techniques such as CT scan and fibre-optic endoscopic examination are not available, clinical examination supported with plain chest X-ray could help to assess visceral involvement.

## 5. Conclusion

KS in Lagos follows the same epidemiologic trend as other centers in Nigeria, with an increasing incidence in this era of HIV/AIDS. Most cases are mucocutaneous and occur at multiple sites, with the lower limbs being the commonest site of involvement.

## Figures and Tables

**Table 1:** Relative frequency of Kaposi sarcoma.

Year	Total number of malignancies	Cases confirmed histologically	Cases diagnosed clinically	Total number of KS cases
2000	239	2	0	2
2001	317	1	0	1
2002	296	1	0	1
2003	274	3	0	3
2004	281	1	2	3
2005	226	3	22	25
2006	411	2	13	15
2007	438	2	19	21
2008	569	5	5	10
2009	559	12	14	26
2010	572	13	14	27
2011	634	6	9	15
2012	621	11	10	21
2013	646	5	7	12
Total	6,083	67	115	182

**Table 2:** Distribution of mucocutaneous KS lesions.

Anatomic site	Patch/plaque	Nodule	Patch/nodule	Ulcers	Total
Upper limbs	5	0	0	0	5
Lower limbs	18	9	4	5	36
Head	8	0	0	0	8
Trunk/genitals	7	0	0	0	7
Multiple sites	70	20	4	4	108
Multiple sites (including viscera)	6	3	1	1	11
Total	114	32	9	10	165
